# Does targeting manual therapy and/or exercise improve patient outcomes in nonspecific low back pain? A systematic review

**DOI:** 10.1186/1741-7015-8-22

**Published:** 2010-04-08

**Authors:** Peter Kent, Hanne L Mjøsund, Ditte HD Petersen

**Affiliations:** 1 Spine Centre of Southern Denmark, Ringe, Denmark; 2 Spine Research Centre, Institute of Regional Health Services Research, University of Southern Denmark, Odense, Denmark; 3 Department of Physiotherapy, University College Lillebælt, Odense, Denmark

## Abstract

**Background:**

A central element in the current debate about best practice management of non-specific low back pain (NSLBP) is the efficacy of targeted versus generic (non-targeted) treatment. Many clinicians and researchers believe that tailoring treatment to NSLBP subgroups positively impacts on patient outcomes. Despite this, there are no systematic reviews comparing the efficacy of targeted versus non-targeted manual therapy and/or exercise. This systematic review was undertaken in order to determine the efficacy of such targeted treatment in adults with NSLBP.

**Method:**

MEDLINE, EMBASE, Current Contents, AMED and the Cochrane Central Register of Controlled Trials were electronically searched, reference lists were examined and citation tracking performed. Inclusion criteria were randomized controlled trials of targeted manual therapy and/or exercise for NSLPB that used trial designs capable of providing robust information on targeted treatment (treatment effect modification) for the outcomes of activity limitation and pain. Included trials needed to be hypothesis-testing studies published in English, Danish or Norwegian. Method quality was assessed using the criteria recommended by the Cochrane Back Review Group.

**Results:**

Four high-quality randomized controlled trials of targeted manual therapy and/or exercise for NSLBP met the inclusion criteria. One study showed statistically significant effects for short-term outcomes using McKenzie directional preference-based exercise. Research into subgroups requires much larger sample sizes than traditional two-group trials and other included studies showed effects that might be clinically important in size but were not statistically significant with their samples sizes.

**Conclusions:**

The clinical implications of these results are that they provide very cautious evidence supporting the notion that treatment targeted to subgroups of patients with NSLBP may improve patient outcomes. The results of the studies included in this review are too patchy, inconsistent and the samples investigated are too small for any recommendation of any treatment in routine clinical practice to be based on these findings. The research shows that adequately powered controlled trials using designs capable of providing robust information on treatment effect modification are uncommon. Considering how central the notion of targeted treatment is to manual therapy principles, further studies using this research method should be a priority for the clinical and research communities.

## Background

'Identifying what treatment works best for whom' [1] in low back pain has been an on-going aim of clinicians and has been a research priority over the last decade [2]. Central to that aim is the notion that targeting treatment to subgroups of people with low back pain might improve patient outcomes and increase health system efficiency. If this aim were achieved, the impact would be widespread, as back pain affects most people at some point in their lives [3] and the consequent health care, community and personal costs are considerable [4].

However, a definitive diagnosis is not possible in 80% of low back pain and is most accurately labelled 'nonspecific low back pain' (NSLBP). Perhaps due to this diagnostic imprecision, there is an ongoing debate about the best treatment for NSLBP [5] and considerable variability in its management across clinical disciplines. For some clinical disciplines, back pain is a frequent reason for people who seek their assistance [6], especially for the primary care professions that practise manual therapy. Manual therapy treatment usually involves manual techniques (mobilization, manipulation and traction) and exercise [7] and these key treatment approaches are recommended in recent international guidelines for the management of low back pain [5,8,9].

A central element in the current debate about best practice management of low back pain is the efficacy of targeted versus generic (non-targeted) treatment. Many primary care clinicians (chiropractors, general practitioners, physiotherapists and osteopaths) resist the notion that non-targeted treatment is appropriate. In clinical practice, they observe highly variable patient presentations and most believe that targeting treatment to people with particular patterns of symptoms and signs (treatment effect modifiers) results in better patient outcomes [10]. Treatment decisions are influenced by this belief but there is little agreement about what symptoms and signs are important treatment effect modifiers [11] and some argue that non-targeted treatment may be equally effective [12,13]. Research that adequately addresses the validity of these disparate approaches to the care of NSLBP is fundamental in order to break the logjam that currently inhibits consensus regarding best practice.

The design of a controlled trial traditionally applies different treatment to two or more groups randomized from a population sample. An assumption is that these groups are similar at baseline on all variables likely to influence outcome and, therefore, any difference in outcome is due to one treatment being more effective than another. However, if the subgrouping approach is correct and important treatment modifiers do occur in NSLBP, trials using this traditional research design may not recognize the heterogeneity of treatment response in participants. This could result in potential treatment effects being diluted by subgroups of people who are unlikely to respond. Due to differential sampling, this unrecognized heterogeneity may also result in contradictory results from replication studies.

Most controlled trials of manual therapy or exercise for NSLBP have been performed using a 'non-targeted treatment' design. Systematic reviews summarizing the results of these trials conclude that these treatments result in better patient outcomes than if no treatment is given, but no particular treatment is clearly better than any other and all produce modest treatment effects [14-17]. On the other hand, clinicians convinced of the benefits of targeted treatment are supported by preliminary evidence that treatment targeted to empirically-derived subgroups of patients with NSLBP can improve patient outcomes and reduce the costs of care [18-21].

There are no systematic reviews comparing the efficacy of targeted versus non-targeted manual therapy and/or exercise. For a systematic review to summarize this evidence, the included trials must contain a clinical prediction rule that seeks to subgroup NSLBP based on treatment modifiers and match treatment to these subgroups. In theory, treatment efficacy may vary depending on the prediction rule used to identify the target population. Therefore, the clinical prediction rule/treatment combinations being tested should be clearly identified in systematic reviews of targeted treatment.

The potential for spurious findings is present at all phases of subgrouping research. Trials in which *post-hoc *analysis was performed in order to identify treatment modifiers (hypothesis-setting studies) provide a lower level of evidence than those where the clinical prediction rule is clearly identified before the start of the trial (hypothesis-testing studies) [22].

There is an important distinction between prognostic factors and treatment effect modifiers. Prognostic factors are symptoms and signs that indicate likely outcomes regardless of treatment. Treatment effect modifiers are symptoms and signs that indicate likely response to a specific treatment. Correct analysis of a clinical prediction rule/treatment effect involves a test of interaction that allows differentiation between prognostic effects and treatment effect modifiers [22,23]. Systematic reviews of targeted treatment need to report this interaction and describe when it is not possible for it to be identified.

The aim of this systematic review was to determine the efficacy of targeted manual therapy and/or exercise on pain and activity limitation in adults with NSLBP.

## Methods

### Types of studies

Inclusion criteria were randomized controlled trials (RCT) comparing targeted manual therapy and/or exercise interventions to non-targeted interventions in NSLBP. They needed to be hypothesis-testing studies and, due to a lack of translation resources, published in English, Danish or Norwegian.

Some studies of targeted treatment investigate simple clinical prediction rules that predict treatment response to one treatment [19] and others investigate more complex clinical reasoning (a subgroup system) that predict treatment response to a number of treatments [18]. For simplicity, in this review the term 'clinical prediction rule' refers to both situations.

Many RCTs that aim to investigate targeted treatment use two-group designs that, due to confounding by different treatments in the two groups or by the research design used, do not provide robust information on treatment effect modification. That is, they are not comparing one targeted treatment with the same treatment when untargeted or they compare two different treatments using methods that do not allow one to determine whether subgrouping is important [24]. Some recent RCTs have overcome this confounding effect by using a more precise design in which the two treatment groups are accompanied by a clinical prediction rule covariate [19]. This covariate allows the identification of the treatment effect in the people who were positive on the prediction rule and the treatment effect in the people who were negative on the prediction rule. This RCT design does provide robust information on treatment effect modification [23] and, in this review, such a study was classified as a 'two-group plus subgroup covariate RCT'.

A second RCT design that is also capable of providing information on the effect of treatment targeted to subgroups is a design that includes more than two treatment groups and tests multiple clinical prediction rules (a subgroup system). In this design, randomization to multiple treatment groups is blind to prediction rule status and the people in each treatment group are a mixture of those who are rule-positive and those who are rule-negative. *Post-hoc *unblinding of rule status allows the identification of the effect of rule-matched and rule-unmatched treatment. In this review, such a study was classified as a'multi-arm subgroup-system RCT'. A disadvantage of this design is that, usually, only the effect size for the whole subgroup system is reported and, therefore, it is not possible to determine if the treatment modifier effects across the subgroups vary - possibly being important for some treatment subgroups in the system and not for others.

Therefore, an additional inclusion criterion for this review was that RCTs needed to be either a 'two-group plus subgroup covariate RCT' or a 'multi-arm subgroup-system RCT'. Exclusion criteria were: observational studies and uncontrolled studies; studies comparing non-targeted interventions; and studies comparing two targeted interventions.

### Types of participants

Participants needed to be experiencing NSLBP, but they could not be pregnant. Arbitrarily, more than 85% of participants needed to be aged 18 years or over. Trials containing people with both low back pain and leg pain were included if at least 85% of the participants had no symptoms or signs of neurocompression (numbness, pins and needles or lower limb muscle weakness) or 'sciatica'. Studies containing participants with specific low back pain (for example, fracture, infection, cancer or inflammatory arthritis) were excluded.

Low back pain was defined as pain occurring below the lower ribs and above the gluteal folds, including the buttocks. Using the Cochrane Back Review Group criteria [25], the duration of back pain was categorized as acute (less than 6 weeks), sub-acute (6 - 12 weeks) and chronic (greater than 12 weeks). Trials with at least 85% of participants whose duration of pain was in one of these temporal categories were only classified under that category.

### Types of intervention

Mobilization, manipulation and traction were classified as 'manual therapy' and were classified as 'exercise' if they included therapeutic exercise [7]. Included studies had to report sufficient data in order to determine the size of the effect attributable to the targeted therapy, including point estimates and measures of variability. The clinical prediction rule used to target the intervention had to have been clearly identified before the trial commenced (hypothesis-testing studies). Trials in which *post-hoc *analysis was performed in order to identify the responders to the intervention (hypothesis-setting studies) were excluded. The clinical prediction rule had to be concordant with the intervention. For example, a trial of 'McKenzie exercises' which contained no assessment of directional preference would have been excluded, as directional preference is central to the clinical reasoning used to determine McKenzie exercise prescription.

### Types of outcome measures

Self-reported pain and activity limitation were the outcome measures. Results were defined as short-term if they were measured less than 3 months after randomization, intermediate-term when between 3 months and 1 year and long-term when greater than 1 year. Where outcomes were measured at multiple time points within these time frames, arbitrarily the outcomes closest to 6 weeks, 6 months and 18 months were used.

### Data sources and search strategy

The electronic databases MEDLINE, EMBASE, Current Contents, AMED and The Cochrane Central Register of Controlled Trials were searched from inception to February 2009. Reference and citation tracking of included articles were performed. A sensitive search strategy was used which was based on that recommended by the Cochrane Back Review Group [25] and is available on request from the first author (PK).

### Selection of studies for inclusion and assessment of method quality

In order to clarify any misinterpretation, the inclusion criteria form was independently piloted on five eligible abstracts by the reviewers (HM, DP and PK). The method quality criteria recommended by the Cochrane Back Review Group [25] were adapted for this review of targeted treatment (see Appendix). All reviewers independently piloted these criteria on three excluded papers. The screening on title and abstract, screening of retrieved articles, method quality assessment and data extraction were independently performed by two reviewers (HM and DP). Any disagreement was resolved by discussion, including a third independent reviewer if required (PK). Assessment of method quality was not blinded to trial authors, institution or journal.

A high quality trial was defined as a trial that obtained, at a minimum, a 'yes' score for randomization, allocation concealment, outcome assessor blinding and also a 'yes' score for any three of the other method quality criteria [25]. As this was a review of targeted manual therapy and/or exercise, 'blinded to the intervention' was defined as inadequate information for the patient or clinician to know whether or not the intervention received was aligned with the specific clinical prediction rule being tested.

### Data extraction

Relevant data were independently extracted by two of the reviewers (HM and DP) using a standardized form that included: quality criteria; participant characteristics; trial characteristics; description of interventions; the decision rules used to target the manual therapy or exercise; and point estimates and measures of variability for outcomes. The data extraction form was pilot-tested on three excluded trials. If necessary, the trial authors were contacted for additional information [18,19,21,26].

### Data synthesis

The group means and standard deviations (SD) were extracted for each comparison on each available outcome measure. Where a SD had to be calculated from a confidence interval, it was calculated using the method described in the Cochrane Handbook (v4.2.5 p117) [27].

All the included trials measured activity limitation using the Oswestry Disability Index [28] or the Roland-Morris Disability Questionnaire [29]. These two assessment instruments attempt to measure the same underlying construct of activity limitation and have been shown to display similar responsiveness [30,31]. All the included trials that measured the effects on pain used a Visual Analogue Scale or Numerical Rating Scale. For the purposes of this systematic review, data derived using either of these activity limitation questionnaires were treated as being comparable and data from either of these measures of pain intensity as being comparable. All pain, activity limitation and patient satisfaction scores were converted to a 0-100 scale.

### Calculating the efficacy of targeting treatment

The mean and SD for each group, for each comparison, at each outcome period (if available) were entered into Cochrane Collaboration Revman (v5.0.2) software to calculate mean effect (mean difference) [32]. Where the outcomes of two treatment groups were combined to create a comparison treatment [21] an *n*-weighted mean and SD were calculated. The direction of all the reported results was standardized so that effects favouring targeted treatment were positive scores. Meta-analysis was not performed due to the clinical diversity and methodological variability of the included studies.

In studies where individual patient data are available, the appropriate statistical analysis needed to differentiate between treatment modifier effects and prognostic effects in a targeted treatment is a test of interaction, often a form of ANOVA [22,23,26]. This is important, as not identifying the prognostic effects in a clinical prediction rule will lead to an over-estimate of targeted treatment efficacy. However, it is rare that authors of systematic reviews have access to individual patient data and, therefore, reviewers of results from targeted treatment studies usually only have access to group summary outcome data. Mark Hancock (personal communication, 2009) recommends that, in circumstances where only study level data are available, the following formula be used to determine the interaction between treatment modifier and treatment allocation:

((people prediction rule-positive and received rule-positive treatment) - (prediction rule-positive and comparison treatment)) - ((prediction rule-negative and rule-positive treatment) - (prediction rule-negative and comparison treatment))

**Figure 1:**
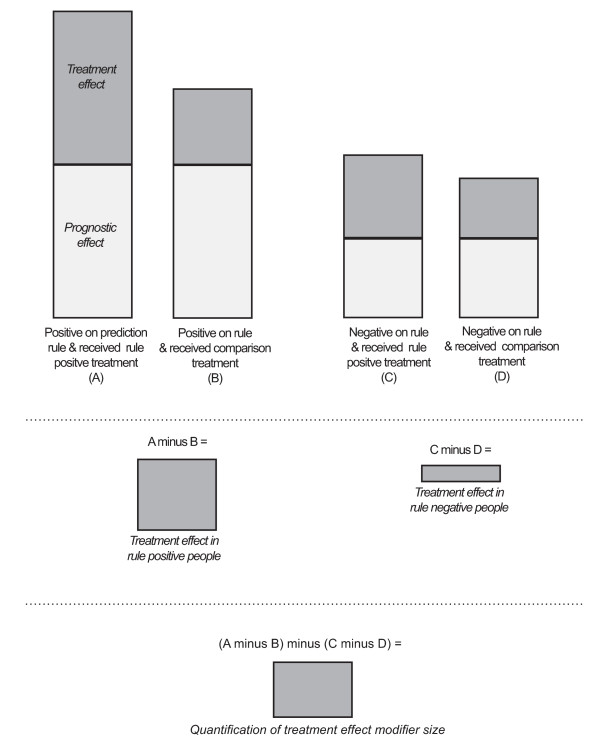
**Diagrammatic illustration of how treatment effect modifier size is isolated from prognostic effects using the 'Hancock formula'**.

The first (double bracketed) part of this formula determines the treatment effect in people who are positive on the clinical prediction rule and who, therefore, share the same prognostic effect as the prediction rule. The second (double bracketed) part of the formula determines the treatment effect in people who are negative on the clinical prediction rule and who also share the same prognostic effect of the prediction rule. The product of the formula is an estimate of the 'treatment effect modifier size' (treatment effect in rule-positive people minus treatment effect in rule-negative people) when the prognostic effect of the prediction rule has been removed. This process is illustrated in Figure 1. In the current review, this Hancock formula was used whenever these data from a 'two-group plus subgroup covariate RCT' were available [19,26]. In this review, the term 'subgroup system effect size' is used to describe the additional effect of subgroup-matched treatment compared with non-subgroup-matched treatment when identified in a 'multi-arm subgroup system RCT'.

To aid the interpretability of the results, the total improvement (clinical course) of the targeted group from baseline was displayed diagrammatically as a proportion of its baseline score. Two components of that improvement were displayed: (a) the proportion of that change attributable to the additional effect of targeting treatment using the clinical prediction rule; and (b) the proportion of that change attributable to other reasons (natural history, nonspecific treatment effects and the likely improvement had this group received the comparison treatment).

## Results

### Search yield

**Figure 2:**
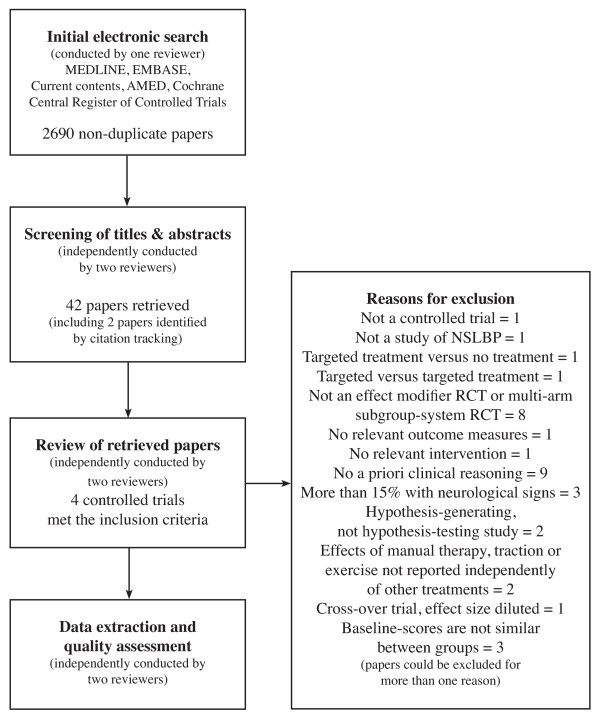
**Review flow chart**.

A flow chart (Figure 2) documents the selection process of trials included in the review. Four studies met the inclusion criteria [18,19,21,26]. The characteristics of the included studies are shown in Table 1. The reasons for the exclusion of other trials retrieved in full text are noted in Table 2.

**Table 1:** Characteristics of included studies

RCT	**Brennan 2006**[18]**(Subacute NSLBP)**	**Childs 2004**[19]**(Acute NSLBP)**	**Hancock 2008**[26]**(Acute NSLBP)**	**Long 2004**[21]**(Chronic NSLBP)**
**Inclusion criteria**	Age 18-65 years. Low back pain (LBP) of less than 90 days with or without referral into the lower extremity, and an Oswestry disability score ≥ 25%.	Age 18-60 years. Primary symptom of LBP, with or without referral into the lower extremity, Oswestry disability score of at least 30%.	LBP of < 6 weeks duration, causing moderate pain and moderate disability (measured by adaptations of items 7 and 8 of the SF-36)	Age 18-65 years. LBP with or without leg symptoms and with or without a neurological sign. Demonstrating a directional preference.
**Exclusion criteria**	A visible lateral shift or acute kyphotic deformity, signs of nerve root compression, red flags indicating a serious pathology, an inability to reproduce any symptoms with lumbar spine active range of motion (AROM) or palpation, pregnancy, prior surgery to the lumbar and/or sacral region.	Patients with red flags for a serious spinal condition, signs consistent with nerve root compression, pregnancy, prior surgery to the lumbar spine or buttock.	Current episode not preceded by a pain-free period of at least one month in which no care was provided, known or suspected serious spinal pathology, nerve root compromise, currently receiving non-steroidal anti-inflammatory drugs or spinal manipulative therapy, surgery within the preceding 6 months, contraindication to paracetamol, diclofenac or spinal manipulative therapy.	Cauda equina syndrome. Two or more neurological signs. Spinal fractures. Post-surgical. Off work for one year or more due to LBP. Medical causes (for example, severe osteoporosis, inflammatory or infectious conditions). Uncontrolled medical conditions (for example, diabetes, angina, hypertension). Pregnancy. Inability to read English. Patients with prior knowledge of, or specific physician referral for, the McKenzie method. No directional preference.
**Clinical prediction rule**	Delitto Treatment Based Classification	Flynn manipulation prediction rule	Flynn manipulation prediction rule	McKenzie directional preference-based exercise
**Targeted treatment**	Mobilization (low amplitude), manipulation (thrust), exercise (AROM, McKenzie or strengthening and stabilization) *n *= 50	Manipulation (thrust), Exercise (ROM) *n *= 70	Mobilization (mostly low velocity spinal mobilization, but 5% received manipulation) *n *= 114	Exercise (McKenzie directional preference exercises) *n *= 70
**Non-targeted treatment**	Mobilization (low amplitude), manipulation (thrust), exercise (AROM, McKenzie or strengthening and stabilization) *n *= 73	Exercise (stabilization, low-stress aerobic, strengthening) *n *= 61	Sham mobilization (detuned ultrasound) *n *= 121	Exercise (exercises opposite to directional preference or non-directional exercises) *n *= 131
**Outcomes**	Oswestry Disability Index	Oswestry Disability Index	Roland Morris Disability Questionnaire, Pain Numerical Rating Scale	Roland Morris Disability Questionnaire, Pain Visual Analogue Scale

NSLBP, nonspecific low back pain.

**Table 2:** Reasons for excluding retrieved studies

Study	Reason for exclusion (studies may have also met other exclusion criteria)
Browder DA, *et al*. (2007)[37]	Not a two-group plus subgroup covariate RCT or multi-arm subgroup system RCT
Cairns MC, *et al*. (2006)[38]	Not a trial of targeted versus non-targeted manual therapy or exercise
Celestini M, *et al*. (2005)[39]	Not a trial of targeted versus non-targeted manual therapy or exercise
Cherkin DC, *et al*. (1998)[40]	Not a trial of targeted versus non-targeted manual therapy and/or exercise
Childs JD, *et al*. (2003)[41]	Not an RCT
Chiradejnant A, *et al*. (2002)[42]	Not a two-group plus subgroup covariate RCT or multi-arm subgroup-system RCT
Chiradejnant A and Kanlayanaphotporn R (2005)[43]	Conference abstract only
Chiradejnant A, *et al*. (2003)[44]	More than 15% with neurological signs
Clare HA, *et al*. (2007)[45]	No relevant outcome measures
Descarreaux M, *et al*. (2002)[46]	Not a two-group plus subgroup covariate RCT or multi-arm subgroup system RCT
Elnaggar IM, *et al*. (1991)[47]	Not a trial of targeted versus non-targeted manual therapy and/or exercise
Erhard, RE, *et al*. (1994)[48]	Targeted versus targeted treatment
Fritz JM, *et al*. (2003)[20]	Not a two-group plus subgroup covariate RCT or multi-arm subgroup system RCT
Fritz JM, Whitman JM, Childs JD. (2005)[49]	Post-hoc analysis (hypothesis-setting)
Fritz JM, *et al*. (2007)[50]	More than 15% with neurological signs
Geisser ME, *et al*. (2005)[51]	Not a two-group plus subgroup covariate RCT or multi-arm subgroup system RCT
Gillan MG, *et al*. (1998)[52]	Not NSLBP
Goodsell M, *et al*. (2000)[53]	Targeted versus no treatment
Greenman PE (1996)[54]	Not an RCT
Hough E, *et al*. (2007)[55]	Not a trial of targeted versus non-targeted manual therapy and/or exercise
Konstantinou K, *et al*. (2007)[56]	Cross-over trial, effect size diluted
Mayer JM, *et al*. (2005)[57]	Not a trial of targeted versus non-targeted manual therapy and/or exercise
Miller ER, *et al*. (2005)[58]	Baseline-scores are not similar between groups (*T*-test)
Monticone M, *et al*. (2004)[59]	Effects of manual therapy, traction or exercise not reported independently of other treatments
Mujic, SE, *et al*. (2004)[60]	Not a trial of targeted versus non-targeted manual therapy and/or exercise
Newton WP (1995)[61]	Not an RCT
North American Spine Society Board of Directors (2003)[62]	Not an RCT
O'Brien N, *et al*. (2006)[63]	Not a trial of targeted versus non-targeted manual therapy and/or exercise
O'Sullivan PB, *et al*. (1997)[64]	Not a two-group plus subgroup covariate RCT or multi-arm subgroup system RCT
Petersen T, *et al*. (2002)[65]	Not a two-group plus subgroup covariate RCT or multi-arm subgroup system RCT
Petersen T, *et al*. (2007)[66]	Not a two-group plus subgroup covariate RCT or multi-arm subgroup system RCT
Riipinen M, *et al*. (2005)[67]	Hypothesis-generating study, not hypothesis-testing study
Rossignol M, *et al*. (2000)[68]	No relevant intervention
Schenk RJ, *et al*. (2003)[69]	More than 15% with neurological signs
Skikiæ EM, *et al*. (2004)[70]	Not a trial of targeted versus non-targeted manual therapy and/or exercise
Spratt, KF, *et al*. (1993)[71]	Effects of manual therapy, traction or exercise not reported independently of other treatments
Stankovic R, Johnell O (1990)[72]	Not a trial of targeted versus non-targeted manual therapy and/or exercise
Sweetman BJ, *et al. (*1993)[73]	Not a trial of targeted versus non-targeted manual therapy or exercise
Wright A, *et al*. (2005)[74]	Not a trial of targeted versus non-targeted manual therapy and/or exercise

NSLBP, nonspecific low back pain; RCT, randomized controlled trials.

### Quality assessment

The quality assessment scores for the included studies are shown in Table 3. The median quality assessment sum score was 8, with a range of 7 to 10. All four RCTs met the criteria of the Cochrane Back Review Group [25] for high quality studies.

**Table 3:** Method quality.

	Method criteria												
	Randomization	Concealed allocation	Baseline equivalence	Patient blinding	Clinical blinding	Outcome blinding	Co-interventions	Compliance	Dropouts	Outcome timing	Intention to treat	Score	High quality*
Brennan 2006[18]	√	√	X	√	√	√	**?**	X	X	√	√	7	Yes
Childs 2004[19]	√	√	√	√	√	√	X	?	√	√	√	9	Yes
Hancock 2008[26]	√	√	√	√	X	√	√	√	√	√	√	10	Yes
Long 2004[21]	√	√	√	X	X	√	√	X	√	?	√	7	Yes

*Quality of trials: high quality defined as a trial that obtains, at a minimum, a 'yes' score for randomization, concealed allocation, blinded outcome assessment and a yes' score for any three of the other method quality criteria.

### Effects of targeting treatment

The included studies investigated a total of three clinical prediction rules for targeting treatment. These are summarised in Table 4 and were the McKenzie directional preference-based exercise [33], the Delitto Treatment Based Classification method [34] and the Flynn manipulation prediction rule [35].

**Table 4:** Summary of clinical prediction rules.

**Brennan 2006**[18]	*Delitto Treatment-Based Classification system* Baseline examination data were used to classify participants into one of *three classification subgroups: ***Specific exercise**: centralize with two or more movements in the same direction (that is, Flexion or extension) *or *centralize with a movement in one direction and peripheralize with an opposite movement. **Manipulation**: onset of symptoms <16 days *and *no symptoms distal to the knee. **Stabilization**: at least three of the following: Average **straight leg raise range of movement **>91 degrees, positive Prone Instability Test, positive aberrant lumbar spine movement, age <40. *(Traction was a potential fourth group, but was not included in this study)*.
**Childs 2004**[19]	*Flynn manipulation prediction rule* Patients were classified as positive (likely to respond to manipulation) if they met at least four of these five criteria: Duration of current episode of symptoms less than 16 days, location of symptoms not extending distal to the knee, score on the Fear Avoidance Beliefs Questionnaire (work subscale) less than 19 points, at least one lumbar spine segment judged to be hypomobile, at least one hip with more than 35° of internal rotation range of motion.
**Hancock 2008**[26]	*Flynn manipulation prediction rule* As above (Flynn 2003)[35].
**Long 2004**[21]	*McKenzie directional preference-based exercise* Patients were classified as having an extension, flexion or lateral directional preference.

The mean effects (mean difference) for all the target treatments are shown in Table 5. Of the 10 treatment/outcome combinations that were reported across all the studies, using the statistical methods employed in this review, two (20%) showed mean effects that were statistically significant (*P *< 0.05) and both were from the same trial [21]. For reference, the means and SD for the groups in each study are listed in Additional file 1.

**Table 5:** Effects of target treatment

Outcomes	Mean duration of pain	Three way test of interaction statistically significant*	Mean effect of targeting treatment (95% confidence interval) (0-100 scale) (positive result favours targeted treatment) Bolded scores are statistically significant
**McKenzie directional preference-based exercises**			
***Short term activity limitation***			
Directional preference matched exercises versus non-directional preference exercises (Long *et al*. 2004)[21]	Chronic	NA^†^	**16.95 [8.74, 25.16]** *P *= 0.000, *n *= 201
***Short term pain***			
Directional preference matched exercises versus non-directional preference exercises (Long *et al*. 2004)[21]	Chronic	NA	**19.80 [14.34, 25.26]** *P *= 0.000, *n *= 201

**Delitto Treatment-Based Classification**			
***Short term activity limitation***			
Treatment matched to classification vs. treatment unmatched to classification (Brennan 2006)[18]	Sub-acute	Yes	5.60 [-0.49, 11.69] *P *= 0.070, *n *= 123
***Long term activity limitation***			
Treatment matched to classification vs. treatment unmatched to classification (Brennan 2006) [18]	Sub-acute	Yes	3.10 [-3.13, 9.33] *P *= 0.330, *n *= 123

**Flynn manipulation rule**			
***Short term activity limitation***			
Manipulation (fitted prediction rule) versus manipulation (didn't fit rule) (Childs *et al*. 2004)[19]	Acute	Yes	8.68 [-1.63, 19.0] *P *= 0.10, *n *= 131
SMT (fitted prediction rule) versus SMT (did not fit prediction rule) (Hancock *et al*. 2008)[26]	Acute	No	-5.50 [-16.09, 5.09] (rule-negative group had better outcome) *P *= 0.310, *n *= 235
***Intermediate term activity limitation***			
Manipulation (fitted prediction rule) versus manipulation (did not fit rule) (Childs *et al*. 2004)[19]	Acute	Yes	3.51 [-6.26, 13.28] *P *= 0.480, *n *= 131
SMT (fitted prediction rule) versus SMT (did not fit prediction rule) (Hancock *et al*. 2008)[26]	Acute	No	**-10.30 [-20.80, 0.20]** (rule-negative group had better outcome) *P *= 0.050, *n *= 235
***Short term pain***			
SMT (fitted prediction rule) versus SMT (did not fit prediction rule) (Hancock *et al*. 2008)[26]	Acute	No	5.60 [-5.48, 16.68] *P *= 0.320, *n *= 235
***Intermediate term pain***			
SMT (fitted prediction rule) versus SMT (did not fit prediction rule) (Hancock *et al*. 2008)[26]	Acute	No	0.40 [-9.84, 10.64] *P *= 0.940, *n *= 235

* Three way test of interaction = (time × treatment group × prediction rule status) test of interaction, such as ANOVA

†NA = not applicable, as a three way test of interaction was not performed.

SMT = Spinal Manipulative Therapy = 97% received spinal mobilization (low velocity techniques) and 5% received manipulation (high velocity).

**Figure 3:**
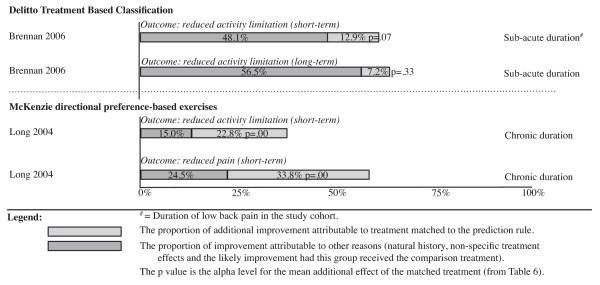
**Improvement in patient outcomes (clinical course) for the targeted treatment group as a proportion of their baseline score**. Evidence of matched treatment effect modification from multi-arm subgroup system randomized controlled trials.

**Figure 4:**
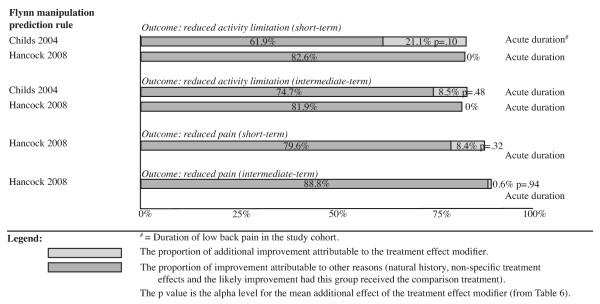
**Improvement in patient outcomes (clinical course) for the targeted treatment group as a proportion of their baseline score**. Evidence of treatment effect modification from two-group plus subgroup covariate randomized controlled trials.

Improvements in patient outcomes (clinical course) for the target treatment group are displayed diagrammatically in Figures 3 to 4 as a proportion of baseline scores. As the capacity to identify treatment modifier effects varies between two-group plus subgroup covariate RCTs and multi-arm subgroup system RCTs, the results from studies with the same RCT design type are presented together.

### McKenzie directional preference-based exercises

A single high quality study investigated McKenzie directional preference-based exercise [21]. It was a multi-arm subgroup system RCT that showed statistically significant improvements in short-term activity and short-term pain limitation due to the matched treatment effect. The size of these effects ranged from 22.8% to 33.8% of baseline scores. As this study only included people with a directional preference, these results are only applicable to people who display a directional preference. However, as it is not clinically congruent to give directional preference exercises to people without a directional preference, this is a reasonable limitation to the generalizability of the study.

The analysis used in this review compared the effect of directional preference exercises with the mean of both comparison groups (the opposite direction exercise group and the non-directional exercise group), but an alternative would have been to use only the opposite direction exercise group as the comparison. As clinicians and patients were not blind to treatment group allocation in this trial, treatment expectation may have inflated the subgroup system effect size in this RCT. Use of only the opposite direction exercise group as the comparison group would have resulted in slightly larger effect sizes (within 3 points on a 0-100 scale). As the outcomes in the opposite direction exercise group may have been biased by a low expectation of treatment outcome, in this review the mean of both comparison groups was chosen as a more conservative estimate of effect.

In summary, a three-way test of interaction was not performed in this study to assess the ability of the prediction rule to identify people who respond to this matched treatment. However, the size of the matched treatment effect was statistically significant for both short-term activity and short-term pain limitation.

### Delitto treatment-based classification

A single high quality study investigated the Delitto treatment-based classification method [18]. The results of this multi-arm subgroup system RCT showed matched treatment effects of 12.9% of baseline scores for short-term activity limitation and 8.5% for short-term pain but neither were statistically significant. In summary, a three-way test of interaction in this study indicated that the ability of the prediction rule to identify people who respond to a matched treatment was statistically significant, but a test of the size of that matched treatment effect was not statistically significant.

### Flynn manipulation prediction rule

Two high quality studies [19,26] investigated the Flynn manipulation prediction rule. Both used the two-group plus subgroup covariate RCT design and were analysed in this review using the Hancock formula.

Childs *et al*. [19] compared targeted spinal manipulation plus range-of-motion exercises with the control treatment of guidelines-based exercise. The results showed a treatment modifier effect size of 21.1% of baseline scores for short-term activity limitation and 8.5% in intermediate-term activity limitation but neither was statistically significant. In summary, a three-way test of interaction in this study indicated that the ability of the prediction rule to identify people who respond to this targeted treatment was statistically significant, but using the statistical methods in this review, the size of that treatment modifier effect was not statistically significant.

Hancock *et al*. [26] compared the results of spinal mobilization with the control treatment of detuned ultrasound. The results showed a treatment modifier effect size of 8.4% of baseline scores for short-term pain and 0.6% in intermediate-term pain but neither was statistically significant. In contrast with Childs *et al*., these results showed 'no treatment modifier effect' on short-term or immediate-term activity limitation and this may reflect a number of factors. One factor may be that the Childs *et al*. RCT used the particular spinal manipulation technique that the Flynn rule was designed for, whereas the Hancock *et al*. RCT was a pragmatic study in which most patients received spinal mobilisation techniques and only 5% received manipulation. Another factor may be the different demographic and cultural settings in which the RCTs occurred. In summary, a three-way test of interaction in the Hancock *et al*. study indicated that the ability of the prediction rule to identify people who respond to a targeted treatment was not statistically significant and a test of the size of any treatment modifier effect was not statistically significant at any outcome time point.

## Discussion

Four RCTs of manual therapy and/or exercise met the inclusion criteria and all were of high method quality. Using the statistical methods in this review, only one study showed statistically significant effect sizes and these were for short-term outcomes [21] following McKenzie directional preference-based exercise. However, there are reasons for caution in the interpretation of these results.

Large effects were only observed in the multi-arm subgroup system RCT of Long *et al*. [21]. Large effects could be defined as being of clinically important size and, in low back pain, clinicians and researchers believe these to be approximately 30% of baseline scores [36]. However, in the Long *et al*. study, patients and clinicians were not blinded to treatment allocation and as one treatment group received exercise that was concordant with prediction rule status (directional preference) and another group received exercise that was opposite to the rule status, an expectation bias may have inflated the effect size. An alternative definition of clinically important effects could be a treatment modifier effect size that results in the cumulative effect being greater than 30% of baseline scores. For example, a treatment modifier effect size of 20% of baseline scores might be clinically useful if, when added to an existing effect of 15%, resulted in the total amount of change being clinically important.

Similarly, large effects were only observed in the RCT that studied people with chronic low back pain [21]. It is possible that subgroup targeted treatment is more effective in people with persistent back pain than in people with a favourable natural history, but the available data in this review are inadequate to test that hypothesis.

All studies that showed, or showed a trend towards, statistically significant effect sizes, did so only for short-term outcomes. It may be that the effects of targeted manual therapy and/or exercise in NSLBP are transient or it may be that the recurrent nature of NSLBP means that treatments that are effective for an episode of pain may, nonetheless, be unable to influence the rate of recurrence or severity of subsequent episodes.

Although the two-group plus subgroup covariate RCT design potentially produces the most accurate estimate of treatment effect modifier size, it requires large samples to obtain adequate power [24]. Only two studies in this review used this two-group plus subgroup covariate RCT design [19,26]. Given their sample sizes, the use of the Hancock formula resulted in increased uncertainty (larger confidence intervals) about the effect size and reinforces the need for adequate power to be included in the design of such RCTs [23]. It is possible that, had the sample sizes been larger in some of the included studies, more effects of targeting treatment would have been statistically significant. For example, the Childs *et al*. study [19] showed a treatment effect modification size of 21.1% of baseline scores for short-term activity limitation but using the statistical method in this review, this result was not significant (*P *= 0.10). However, had the sample size in the study been increased to approximately 211 participants, instead of 131, this result would have been statistically significant. One method to reduce the uncertainty in estimates of the effect of targeted treatment in future trials would be to achieve adequate sample sizes through multi-centre and multi-national collaboration.

Tests of interaction, such a three-way ANOVA of treatment group × prediction rule status × time, can determine whether a clinical prediction rule identifies patients who respond to the target treatment but they do not quantify treatment effect modifier size. Treatment effect modifier size is the difference in effect in rule-positive people compared with rule-negative people at a particular outcome time period. Identifying the treatment effect modifier size requires alternative statistical techniques, such as observing the size of the interaction coefficient from linear regression. As it is uncommon for such results to be reported, systematic reviewers have to use alternative strategies, such as the Hancock formula, to compare effect sizes across studies. It is possible that a prediction rule may, at a statistically significant level, identify people who respond to a target treatment but the treatment effect modifier size at a particular time point is not statistically significant or clinically important. In a multi-arm subgroup system RCT, the equivalent test that identifies the matched treatment effect size is a *T*-test, or a pairwise post-hoc comparison after an ANOVA.

If it is important to investigate whether targeted manual therapy is more useful than non-targeted care, more studies that use this two-group plus subgroup covariate RCT design and tests of interaction should be performed, as they uniquely allow precise identification of treatment effect modifier size for a specific treatment/subgroup combination. Even where statistically significant results of clinically important size are reported in such studies, there remains a need for replication studies, preferably by independent research groups, in order to clarify the stability of findings across samples, care settings and cultures. Considering how central the notion of targeted treatment is to manual therapy principles, it is noteworthy how few high quality, well-designed RCTs have been performed to test hypotheses about the efficacy of targeted treatment.

The strengths of this systematic review are the comprehensiveness of the search, the rigour in the method of analysis and the attempt to address the absence of previously published reviews that quantify the effects of targeted manual therapy and/or exercise on patient outcomes. A limitation of this review is that as only published papers in the English, Danish and Norwegian languages were included, there is potential for the findings to contain cultural and publication bias.

## Conclusions

Four high quality studies were included in this review of targeted manual therapy and/or exercise for NSLBP. Statistically significant effects were rare and when present were only for short-term outcomes. The clinical implications of these results are that they provide very cautious evidence supporting the notion that treatment targeted to subgroups of patients with NSLBP may improve patient outcomes but this notion has yet to be adequately researched. The results of the studies included in this review are too patchy, inconsistent and investigated in samples too small for recommendations for targeting treatment in routine clinical practice to be based on these findings. The research implications are that adequately powered RCTs using designs capable of providing robust information on treatment effect modification are infrequent. Considering how central the notion of targeted treatment is to manual therapy principles, further studies using this research method should be a priority for the clinical and research communities.

## Abbreviations

NSLBP: nonspecific low back pain; RCT: randomized controlled trials; SD: standard deviation.

## Competing interests

The manuscript submitted does not contain information about medical devices or drugs. No benefits in any form have been, or will be, received from a commercial party related directly or indirectly to the subject of this manuscript.

## Authors' contributions

The conception and design of the study and initial draft manuscript were by PK. All authors (PK, HM, DP) were involved in the analysis and interpretation of data, revision of the manuscript and approval of the final manuscript.

## Appendix

### Criteria list for the method quality assessment

A. Was the method of randomization adequate? A random (unpredictable) assignment sequence. Examples of adequate methods are computer generated random number table and use of sealed opaque envelopes. Methods of allocation using date of birth, date of admission, hospital numbers, or alternation will not be regarded as appropriate.

B. Was the treatment allocation concealed? Assignment generated by an independent person not responsible for determining the eligibility of the patients. This person has no information about the persons included in the trial and has no influence on the assignment sequence or on the decision about eligibility of the patient.

C. Were the groups similar at baseline regarding the most important prognostic indicators? In order to receive a 'yes', groups have to be similar at baseline regarding demographic factors, duration and severity of complaints, and value of main outcome measure(s).

D. Was the patient blinded to the intervention? The review author determines if enough information about the blinding is given in order to score a 'yes'. A yes is awarded if the participant was blind to the results of the clinical prediction rule, as the comparison in this review is between the effect of manual therapy or exercise when targeted using this clinical prediction rule versus the effect of the same manual therapy or exercise when not targeted.

E. Was the care provider blinded to the intervention? The review author determines if enough information about the blinding is given in order to score a 'yes'. A yes was awarded if the care provider was blind to whether the manual therapy or exercise was targeted or not, as the comparison in this review is between the effect of treatments when targeted versus the effect of the same treatments when not targeted.

F. Was the outcome assessor blinded to the intervention? The review author determines if enough information about the blinding is given in order to score a 'yes'. A yes is awarded if the outcome assessor was reported to be blinded to whether the intervention was targeted or not, even if the details of how blinding was maintained is not provided.

G. Were co-interventions avoided or similar? Co-interventions should either be avoided in the trial design or be similar between the index and control groups. A yes is awarded if the authors collected data on co-interventions and tested for differences between groups or if a comparable proportion of people in each group reported seeking additional treatment.

H. Was the compliance acceptable in all groups? The review author determines if the compliance to the interventions is acceptable, based on the reported intensity, duration, number and frequency of sessions for both the index intervention and control intervention(s) Acceptable compliance was defined as adherence levels of 80% for short-term follow-ups and 70% for intermediate-term and long-term follow-ups.

I. Was the drop-out rate described and acceptable? The number of participants who were included in the study but did not complete the observation period or were not included in the analysis must be described and reasons given. If the percentage of withdrawals and drop-outs does not exceed 20% for immediate-term and short-term follow-ups, 30% for intermediate-term and long-term follow-ups and does not lead to obvious bias a 'yes' is scored.

J. Was the timing of the outcome assessment in all groups similar? Timing of outcome assessment should be identical for all intervention groups and for all important outcome assessments.

K. Did the analysis include an intention-to-treat analysis? All randomised patients are reported/analysed in the group they were allocated to by randomization for the most important moments of effect measurement (minus missing values) irrespective of noncompliance and co-interventions. When data are missing, acceptable strategies, such as mean substitution, last recorded measurement etc are used in data analysis.

## Pre-publication history

The pre-publication history for this paper can be accessed here:

http://www.biomedcentral.com/1741-7015/8/22/prepub

## Supplementary Material

Additional file 1 Table of mean scores and standard deviations for the groups in each included study. Click here for file
