# Supplementary material for: Does targeting manual therapy and/or exercise improve patient outcomes in nonspecific low back pain? A systematic review
Source: BMC Med. 2010 Apr 8;8:22. doi: 10.1186/1741-7015-8-22 (PMC2873245; doi:10.1186/1741-7015-8-22)
Supplement: Additional file 1 — Table of mean scores and standard deviations for the groups in each included study. [file 1741-7015-8-22-S1.DOC]

**Additional file 1.** **Table of means and SD for the groups in each study**

|  | **Prediction rule matched** | | | **Prediction rule unmatched** | | |
| --- | --- | --- | --- | --- | --- | --- |
|  | **Mean** | **SD** | **n** | **Mean** | **SD** | **n** |
| ***McKenzie directional preference exercises*** | | | | | | |
| **Activity limitation (short-term)** | | | | | | |
| Long et al 2004 | 47.41 | 31.48 | 70 | 64.36^#^ | 21.11^*^ | 131 |
| **Pain (short-term)** | | | | | | |
| Long et al 2004 | 25.1 | 19.6 | 70 | 44.9^#^ | 17.2^*^ | 131 |
| ***Delitto Treatment-Based Classification*** | | | | | | |
| **Activity limitation (short-term)** | | | | | | |
| Brennan et al 2006 | 16.9 | 17.3 | 50 | 22.5 | 16.4 | 73 |
| **Activity limitation (long-term)** | | | | | | |
| Brennan et al 2006 | 15.7 | 17.2 | 50 | 18.8 | 17.5 | 73 |

^#^ n-weighted mean, ^*^ n-weighted standard deviation

|  |  | **Rule-positive treatment** | | **Comparison treatment** | |
| --- | --- | --- | --- | --- | --- |
|  |  | **Fitted prediction rule** | **Didn’t fit prediction rule** | **Fitted prediction rule** | **Didn’t fit prediction rule** |
| ***Flynn manipulation prediction rule*** | | | | | |
| **Short-term activity limitation** | | | | | |
| Childs 2004 | Mean | 7.52 | 22.72 | 22.08 | 28.6 |
|  | SD | 7.47 | 17.56 | 14.71 | 18.92 |
|  | n | 23 | 47 | 24 | 37 |
| Hancock 2008 | Mean | 10.00 | 12.25 | 10.17 | 17.92 |
|  | SD | 16.62 | 20.25 | 16.23 | 25.29 |
|  | n | 68 | 50 | 71 | 46 |
| **Intermediate-term activity limitation** | | | | | |
| Childs 2004 | Mean | 7.44 | 17.78 | 20.3 | 27.14 |
|  | SD | 12.44 | 16.72 | 14.1 | 18.88 |
|  | n | 23 | 47 | 24 | 37 |
| Hancock 2008 | Mean | 9.38 | 8.75 | 6.00 | 15.67 |
|  | SD | 16.90 | 17.90 | 15.92 | 26.44 |
|  | n | 68 | 50 | 71 | 46 |
| **Short-term pain** | | | | | |
| Hancock 2008 | Mean | 8.1 | 19.2 | 11.1 | 16.6 |
|  | SD | 13.85 | 22.94 | 16.95 | 20.14 |
|  | n | 68 | 50 | 71 | 46 |
| **Intermediate-term pain** | | | | | |
| Hancock 2008 | Mean | 7.1 | 11.2 | 8.5 | 12.2 |
|  | SD | 15.56 | 20.37 | 17.54 | 23.28 |
|  | n | 68 | 50 | 71 | 46 |
